# Src drives the Warburg effect and therapy resistance by inactivating pyruvate dehydrogenase through tyrosine-289 phosphorylation

**DOI:** 10.18632/oncotarget.7159

**Published:** 2016-02-03

**Authors:** Yue Jin, Qingsong Cai, Anitha K. Shenoy, Sangbin Lim, Ying Zhang, Steve Charles, Miriam Tarrash, Xueqi Fu, Sushama Kamarajugadda, Jose G. Trevino, Ming Tan, Jianrong Lu

**Affiliations:** ^1^ Department of Biochemistry and Molecular Biology, UF Health Cancer Center, University of Florida College of Medicine, Gainesville, FL 32610, USA; ^2^ School of Life Sciences, Jilin university, Changchun 130012, China; ^3^ Mitchell Cancer Institute, University of South Alabama, Mobile, AL 36604, USA; ^4^ Department of Surgery, UF Health Cancer Center, University of Florida College of Medicine, Gainesville, FL 32610, USA

**Keywords:** Warburg effect, pyruvate dehydrogenase, Src, reactive oxygen species, chemoresistance

## Abstract

The Warburg effect, which reflects cancer cells' preference for aerobic glycolysis over glucose oxidation, contributes to tumor growth, progression and therapy resistance. The restraint on pyruvate flux into mitochondrial oxidative metabolism in cancer cells is in part attributed to the inhibition of pyruvate dehydrogenase (PDH) complex. Src is a prominent oncogenic non-receptor tyrosine kinase that promotes cancer cell proliferation, invasion, metastasis and resistance to conventional and targeted therapies. However, the potential role of Src in tumor metabolism remained unclear. Here we report that activation of Src attenuated PDH activity and generation of reactive oxygen species (ROS). Conversely, Src inhibitors activated PDH and increased cellular ROS levels. Src inactivated PDH through direct phosphorylation of tyrosine-289 of PDH E1α subunit (PDHA1). Indeed, Src was the main kinase responsible for PDHA1 tyrosine phosphorylation in cancer cells. Expression of a tyrosine-289 non-phosphorable PDHA1 mutant in Src-hyperactivated cancer cells restored PDH activity, increased mitochondrial respiration and oxidative stress, decreased experimental metastasis, and sensitized cancer cells to pro-oxidant treatment. The results suggest that Src contributes to the Warburg phenotype by inactivating PDH through tyrosine phosphorylation, and the metabolic effect of Src is essential for Src-driven malignancy and therapy resistance. Combination therapies consisting of both Src inhibitors and pro-oxidants may improve anticancer efficacy.

## INTRODUCTION

Cancer cells typically exhibit enhanced glucose uptake, glycolysis, and lactate production regardless of oxygen availability, a phenotype known as aerobic glycolysis or the Warburg effect. Increased yield of intermediate glycolytic metabolites fuels macromolecule biosynthesis, thereby providing essential anabolic support to sustain cell proliferation and tumor growth [1, 2]. The end product of glycolysis, pyruvate, is mostly kept away from mitochondrial oxidative metabolism in cancer. Therefore, in cancer cells, mitochondrial oxidation of pyruvate is uncoupled from glycolysis. Because mitochondrial respiration is the major cellular source of reactive oxygen species (ROS) [3], the Warburg metabolic phenotype allows cancer cells to avoid generating excess mitochondrial ROS from pyruvate oxidation, limit cellular oxidative stress, and thus acquire improved metastatic potential [4, 5]. As conventional radiation and chemotherapy kill cancer cells in large part through generation of ROS [6], limiting ROS production also renders cancer cells less susceptible to oxidative stress-induced cell death and hence confers resistance to pro-oxidant therapy [7, 8]. Overall, the Warburg effect promotes tumor growth, metastasis and therapy resistance.

Entry of glycolysis-derived pyruvate into mitochondrial oxidative metabolism is primarily governed by the pyruvate dehydrogenase complex (PDC) in mitochondria. PDC converts pyruvate to acetyl-CoA, which subsequently enters the tricarboxylic acid (TCA) cycle. Therefore, PDC is the gatekeeper enzyme that strategically links glycolysis to mitochondrial oxidation [9]. PDC has a profound impact on whether pyruvate should be oxidized in mitochondria or converted to lactate in cytosol, thereby critically controlling glucose metabolism and oxidative stress. Accumulating evidence suggests that inhibition of PDC is an important contributor to the Warburg effect in cancer cells. According to the current dogma, PDC is principally inactivated by phosphorylation of three specific serine residues on pyruvate dehydrogenase (PDH), the first and most important enzyme component of PDC [9, 10]. Such phosphorylation is catalyzed by pyruvate dehydrogenase kinases (PDKs) [10]. Hypoxia-inducible factors (HIFs), oncoprotein Myc and canonical Wnt signaling can transcriptionally upregulate PDK expression, thereby attenuating PDC activity, pyruvate oxidation and ROS production [11–15]. Consistent with frequent activation of HIF, Myc and Wnt signaling in cancer, PDKs are overexpressed in a variety of human malignancies and contribute to the Warburg effect [5, 16, 17].

While metabolic alterations are common in cancer, the underlying driving forces remain incompletely understood. Only a few oncogenes and tumor suppressors have been shown to rewire cellular metabolism [18, 19]. Src, a non-receptor tyrosine kinase, is the most widely known and the prototypical member of the Src family of tyrosine kinases (SFKs) [20, 21]. SFKs critically transmit signals downstream of cell surface receptors. Many Src family members have been identified as proto-oncogenes, and viral Src is indeed the first identified oncogene [22]. Aberrantly activated SFKs drive a multitude of malignant properties, including cell proliferation, survival, migration, invasion, angiogenesis and metastasis [23–25]. Src also confers resistance to traditional ionizing radiation and chemotherapy [26], as well as to endocrine and anti-HER2 targeted therapies in breast cancer [23], although the underlying mechanisms remain poorly understood. Src overexpression and/or hyperactivation are evident in a wide range of human cancers, and correlate with disease recurrence and adverse prognosis [21,23].

However, the key Src-dependent downstream signaling events contributing to the aggressive and therapy-resistant malignant phenotypes are not clearly elucidated. It was unclear whether and how Src might reprogram metabolism to foster malignancy. Here we found that Src directly phosphorylated PDH and was the major kinase responsible for PDH tyrosine phosphorylation in cancer cells. Tyrosine phosphorylation of PDH suppressed PDH activity, mitochondrial respiration and ROS generation, and increased metastasis and resistance to chemotherapy. The study thus identified a metabolic basis of Src's pro-malignant function.

## RESULTS

### Activation of Src attenuates PDH activity and oxidative stress

**Figure 1:**
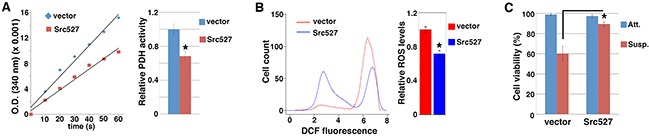
Activation of Src inhibits PDH activity and ROS generation, and confers anoikis resistance MCF10A cells were transduced with lentivirus expressing activated Src527, and subjected to measurement of PDH activity **A.** ROS content **B.** and anoikis sensitivity **C.**. Error bars represent standard deviation (S.D.). *p < 0.05.

Cancer cells may acquire increased metastatic potential by attenuating PDH and mitochondrial oxidative stress [4]. Given Src's pro-metastatic role, we asked whether Src might modulate PDH activity and mitochondrial metabolism. To activate Src, we ectopically expressed Src527, a constitutively active form of Src [27, 28], in MCF10A untransformed mammary epithelial cells through lentiviral transduction. We prepared mitochondrial extracts from control and Src527-expressing cells and measured PDH activity. MCF10A cells expressing activated Src displayed lower PDH activity than control cells (Figure 1A). PDH promotes pyruvate flux into mitochondrial oxidative metabolism, which is a major source of ROS [3]. As expected, Src-activated MCF10A cells showed decreased ROS levels compared with control cells (Figure 1B). SW480 colon cancer cells displayed little intrinsic Src activity [29]. Similar to MCF10A cells, expression of Src527 in SW480 cells also resulted in suppression of PDH activity and ROS content (Supplementary Figure S1). Epithelial cells normally depend on attachment to extracellular matrix for growth and survival, and matrix detachment causes a form of apoptosis known as anoikis [30]. Anoikis is attributed to ROS, which arise from cell detachment [4, 31–34]. Consistently, Src-activated MCF10A cells survived in suspension significantly better than control cells (Figure 1C). Collectively, these results suggest that activation of Src inhibits PDH activity and ROS generation, and confers anoikis resistance.

**Figure 2:**
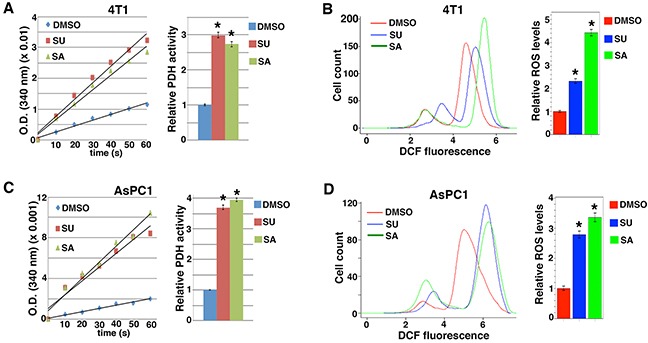
Src inhibitors activate PDH and enhance ROS production 4T1 **A, B.** and AsPC1 **C, D.** cells were treated with indicated Src inhibitors, followed by measurement of PDH activity (A, C). and ROS levels (B, D). SU6656 (SU): 2 μM, Saracatinib (SA): 2 μM. Error bars represent S.D. *p < 0.01.

Conversely, we tested whether inhibition of Src in cancer cells with heightened Src activity might activate PDH and enhance ROS production. Both 4T1 mammary carcinoma cells and AsPC1 pancreatic cancer cells showed hyper-activation of Src [35, 36]. Treatment of these cells with small molecule compounds SU6656 and Saracatinib [37–39], which are selective Src kinase inhibitors, markedly increased PDH activity and ROS levels (Figure 2A–2D). The observations confirm that Src suppresses PDH function and ROS production.

### PDH is tyrosine-phosphorylated in cancer cells in a Src-dependent manner

PDKs inactivate PDH through serine phosphorylation of its E1α subunit (PDHA1) [9, 10]. PDH is predominantly localized in mitochondria. Interestingly, Src family tyrosine kinases such as Src, Lyn, Fyn, and Fgr are also present in or transported into mitochondria as reported by multiple independent laboratories [40–47]. Therefore, we wondered whether Src might inhibit PDH activity through direct tyrosine phosphorylation of PDHA1.

**Figure 3:**
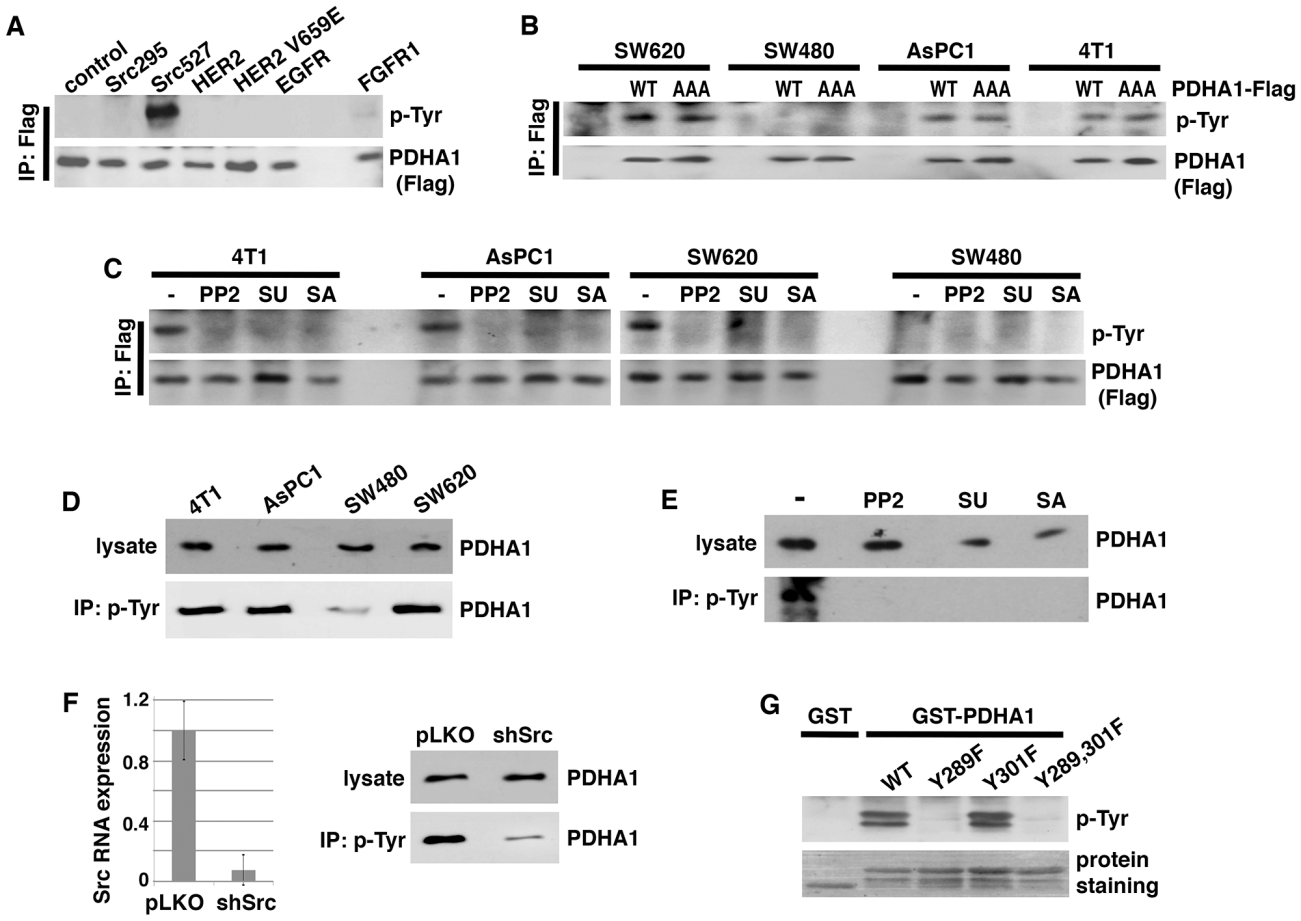
Src is required for PDHA1 tyrosine phosphorylation in cancer cells and directly phosphorylates PDHA1 at Y289 **A.** Src induces PDHA1 tyrosine phosphorylation. PDHA1 with a carboxyl-terminal Flag tag (PDHA1-Flag) was transiently transfected into HEK293 cells together with indicated tyrosine kinases. PDHA1 was immunoprecipitated with anti-Flag antibodies and immunoblotted with anti-phosphotyrosine (p-Tyr) antibodies. **B.** Exogenous PDHA1 is tyrosine-phosphorylated in cancer cells. Indicated cancer cells were transduced with lentivirus expressing wild type (WT) or AAA mutant PDHA1-Flag, followed by immunoprecipitation with anti-Flag antibodies and immunoblotting ith anti-p-Tyr antibodies. AAA mutant PDHA1 lacks PDK phosphorylation sites. **C.** Src inhibitors block tyrosine phosphorylation of exogenous PDHA1 in cancer cells. Indicated cancer cells were transduced with lentiviral PDHA1-Flag. Cells were treated with Src inhibitors PP2 (5 μM), SU (2 μM) or SA (2 μM), then harvested for immunoprecipitation with anti-Flag antibodies and immunoblotting for tyrosine phosphorylation. **D.** Endogenous PDHA1 proteins are tyrosine-phosphorylated in Src-activated cancer cells. Whole cell lysates were prepared from indicated cancer cells, and subjected to either immunoblotting with anti-PDHA1 antibodies or immunoprecipitation with anti-p-Tyr antibodies, followed by immunoblotting with anti-PDHA1. **E.** Src inhibitors abolish tyrosine phosphorylation of endogenous PDHA1. AsPC1 cancer cells were treated with PP2 (5 μM), SA (1 μM) or SU (1 μM), followed by immunoprecipitation with anti-p-Tyr antibodies and immunoblotting with anti-PDHA1 antibodies. **F.** Src is required for tyrosine phosphorylation of endogenous PDHA1. AsPC1 cells were infected with lentiviral vector (pLKO) or shRNA targeting Src (shSrc). Src depletion efficiency was verified by quantitative RT-PCR. Cells were lysed for immunoprecipitation with anti-p-Tyr antibodies, followed by immunoblotting with anti-PDHA1 antibodies. **G.** Src phosphorylates Y289 of PDHA1. WT and tyrosine mutant forms of PDHA1 (aa 281-356) were fused to GST. The purified fusion proteins were incubated with recombinant Src enzyme, followed by immunoblotting with anti-p-Tyr antibodies.

To facilitate the detection of PDHA1 tyrosine phosphorylation, we generated a lentiviral vector expressing PDHA1 with a Flag tag on its carboxyl terminus (PDHA1-Flag). Following transfection into HEK293 cells, PDHA1-Flag proteins were immunoprecipitated with anti-Flag antibodies and immunoblotted with phosphotyrosine-specific antibodies. When expressed alone in HEK293 cells, PDHA1-Flag was not tyrosine-phosphorylated (Figure 3A). However, when co-transfected with constitutively activated Src (Src527), PDHA1 showed strong tyrosine phosphorylation (Figure 3A). By contrast, a kinase-inactive Src295 mutant [48] failed to induce PDHA1 tyrosine phosphorylation, suggesting that Src causes PDHA1 tyrosine phosphorylation through its kinase activity.

Besides Src kinases [40–47], several receptor tyrosine kinases such as EGFR [42], HER2 [49] and FGFR1 [50] have been reported to be located in mitochondria. When co-transfected with PDHA1-Flag, wild-type (WT) and a constitutively active V659E mutant of HER2 [51] as well as EGFR did not lead to PDHA1 tyrosine phosphorylation (Figure 3A). FGFR1 was recently reported to phosphorylate PDHA1 [52]. FGFR1 induced weakly detectable tyrosine phosphorylation of PDHA1 in this assay (Figure 3A).

Based on Src's ability to induce PDHA1 tyrosine phosphorylation, we verified whether PDHA1 was tyrosine-phosphorylated in Src-activated cancer cells. In addition to 4T1 and AsPC1 cells, SW620 colon cancer cells also expressed hyper-activated Src [53]. SW620 cells were derived from lymph node metastasis of the same cancer patient as SW480 cells [54]. We infected these high-Src cells as well as low-Src SW480 cells (negative control) with lentiviral PDHA1-Flag. PDHA1-Flag proteins were immunoprecipitated with anti-Flag antibodies followed by immunoblotting for phospho-tyrosine. Tyrosine phosphorylation of PDHA1-Flag was indeed detected in SW620, 4T1 and AsPC1 cells, but not in SW480 cells (Figure 3B), suggesting that PDHA1 tyrosine phosphorylation correlates with Src activity. To further determine whether tyrosine phosphorylation of PDHA1 might require PDK-mediated serine phosphorylation, we stably expressed the AAA mutant form of PDHA1-Flag that lacked the three serine phosphorylation sites for PDKs through lentiviral infection [4]. PDHA1 AAA mutant also exhibited tyrosine phosphorylation in SW620, 4T1 and AsPC1 cells (Figure 3B), suggesting that PDHA1 tyrosine phosphorylation is independent of PDK-mediated serine phosphorylation.

We next asked whether PDHA1 tyrosine phosphorylation in these cancer cells was dependent on Src. We treated PDHA1-Flag-transduced SW620, 4T1 and AsPC1 cells with chemically distinct Src kinase inhibitors, including PP2, SU6656, and Saracatinib [37–39], and immunoprecipitated PDHA1-Flag to determine the tyrosine phosphorylation status. All three Src inhibitors abolished PDHA1 tyrosine phosphorylation (Figure 3C). This result suggests that Src kinases are essential for PDHA1 tyrosine phosphorylation in these cancer cells.

Based on the observations of exogenous PDHA1, we sought to determine whether endogenous PDHA1 proteins were tyrosine-phosphorylated by Src. The protein levels of endogenous PDHA1 were comparable among SW620, 4T1, AsPC1 and SW40 cells (Figure 3D). We immunoprecipitated tyrosine-phosphorylated proteins from these cells using anti-phospho-tyrosine antibodies, and examined the protein abundance of PDHA1 in the precipitates. Tyrosine-phosphorylated PDHA1 levels in SW480 cells were much lower than those in SW620, 4T1 and AsPC1 cells (Figure 3D). To validate that endogenous PDHA1 tyrosine phosphorylation in the high-Src cells was attributed to Src, we treated AsPC1 cells with Src inhibitors PP2, SU6656, and Saracatinib. Isolation of tyrosine-phosphorylated proteins from treated cells confirmed that all Src inhibitors abolished tyrosine phosphorylation of endogenous PDHA1 (Figure 3E). Finally, we depleted Src in AsPC1 cells using lentiviral short-hairpin RNA (shRNA) (Figure 3F). Depletion of Src did not change the total protein levels of endogenous PDHA1, however, it strongly decreased the abundance of tyrosine-phosphorylated PDHA1 (Figure 3F). Taken together, these results demonstrate that PDHA1 is tyrosine-phosphorylated in cancer cells in a Src-dependent manner.

### Src directly phosphorylates tyrosine 289 of PDHA1

To investigate whether Src might directly phosphorylate PDHA1, we performed *in vitro* kinase assay. Large-scale phospho-proteomics studies have revealed that PDHA1 could be phosphorylated at multiple tyrosine (Y) residues in various normal and tumor cells, with Y289 and Y301 as the most heavily phosphorylated tyrosine sites (http://www.phosphosite.org). Y301 was reported to be the FGFR1 phosphorylation site [52]. We prepared bacteria-expressed recombinant PDHA1 protein that was fused to glutathione S-transferase (GST). We also generated mutant GST-PDHA1 fusion proteins in which Y289 and Y301 of PDHA1 were substituted with phenylalanine (F). The WT and mutant GST-PDHA1 proteins were incubated with recombinant active Src enzyme, followed by immunoblotting with phospho-tyrosine antibodies. Tyrosine phosphorylation of WT and Y301F mutant PDHA1 by Src was readily detected (Figure 3G). However, PDHA1 Y289F mutant completely resisted Src-mediated tyrosine phosphorylation (Figure 3G). These results suggest that Src can directly phosphorylate PDHA1 specifically at Y289. Therefore, PDHA1 is a new substrate of Src.

PDHA1 Y289 is a highly conserved residue (Supplementary Figure S2). To understand the consequence of its phosphorylation on PDH activity, we examined the crystal structure of PDH [10]. PDH-catalyzed decarboxylation of pyruvate requires thiamin diphosphate (ThDP or TPP) [9]. In TPP-bound PDHA1 [10], Arginine (R) 288 is one of the critical TPP-anchoring residues, and Y289 is in close proximity to Aspartic acid (D) 315 (Supplementary Figure S3). Y289 is located at the protein surface and is accessible to enzymes that may modify it. Upon phosphorylation, the bulky phosphoryl group at Y289 will pose a steric clash with D315. The resultant repulsion is expected to affect the positioning of Y289 and hence the neighboring R288, thereby interfering with the binding of TPP and the enzymatic activity. This model is consistent with the observation that Src decreased PDH activity.

### PDHA1 Y289 phosphorylation is essential for Src's metabolic and pro-malignant effects

**Figure 4:**
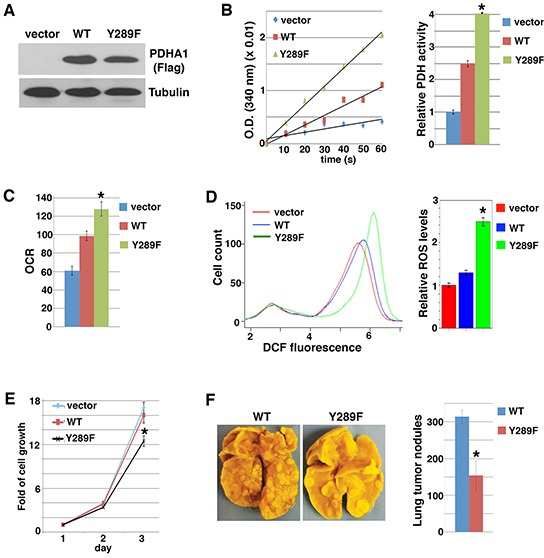
PDHA1 Y289F mutant activates PDH and oxidative metabolism, and reduces cell growth and metastasis 4T1 cells were transduced with lentiviral control vector or PDHA1-Flag (WT or Y289F), followed by analyses of immunoblotting with anti-Flag antibodies **A.** PDH activity **B.** oxygen consumption rate (OCR) **C.** ROS levels **D.** cell growth (living cell number) **E.** and metastatic potential in the experimental metastasis assay (n=5) **F.**. Error bars represent S.D. *p < 0.05.

Activated Src can phosphorylate many substrates implicated in a variety of malignant phenotypes [55]. It was unclear whether PDHA1 Y289 phosphorylation by Src might be biologically significant in regards to Src-mediated oncogenic function. We stably expressed the Src-resistant PDHA1 Y289F mutant in Src-activated cancer cells through lentiviral transduction, and examined whether it reversed Src's effect on metabolism and cell proliferation/survival. When expressed in 4T1 cells (Figure 4A), both WT and Y289F mutant PDHA1 increased PDH activity, but Y289F mutant exhibited a substantially stronger effect than WT PDHA1 (Figure 4B). This result suggests that Src inhibits PDH in large part through PDHA1 Y289 phosphorylation. Consistent with the PDH activity, 4T1 cells expressing Y289F PDHA1 displayed higher oxygen consumption rates and ROS content than those expressing WT PDHA1 (Figure 4C and 4D). Similarly, in SW620 cells, Y289F PDHA1 also led to more robust PDH activation and ROS generation than WT PDHA1 (Supplementary Figure S4A and Supplementary Figure S4B). These results support that PDHA1 Y289 phosphorylation is essential for Src to suppress PDH activity and mitochondrial oxidative metabolism.

Heightened oxidative stress reduces cell growth and viability [7, 8], and sensitizes cancer cells to anoikis as well as suppresses metastasis [4, 5, 32, 56]. Expression of WT PDHA1 in 4T1 and SW620 cells did not significantly affect cell growth, however, expression of Y289F PDHA1 decreased cell proliferation (Figure 4E and Supplementary Figure S4C). As Src activation conferred anoikis resistance (Figure 1C), the viability of SW620 cells was not altered when they were cultured in suspension (Supplementary Figure S4D). But increased cell death was observed in cells expressing Y289F PDHA1 compared with cells expressing WT PDHA1 following cell detachment (Supplementary Figure S4D). Because metastatic cancer cells need to survive the rigors of the dissemination journey, resistance to anoikis is a prerequisite for metastasis [57]. 4T1 cancer cells are highly metastatic [58]. In the experimental metastasis assay, 4T1 cells expressing PDHA1 Y289F generated fewer lung metastases than cells expressing WT PDHA1 after injection into tail vein of recipient mice (Figure 4F). Collectively, these results suggest that Src's pro-malignant activity is at least in part attributed to its metabolic reprogramming effect through PDHA1 Y289 phosphorylation.

**Figure 5:**
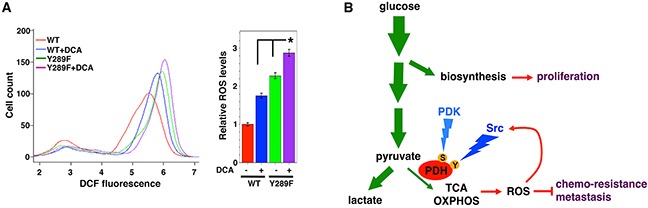
Src and PDK independently suppress ROS production **A.** Inhibition of both Src and PDKs additively increases ROS levels. 4T1 cells transduced with WT or Src-resistant Y289F PDHA1 were treated with DCA (10 mM), followed by ROS measurement. Error bars represent S.D. *p < 0.05. **B.** A simplified view of glucose metabolic reprogramming in cancer. During glycolysis in cancer cells, a significant portion of glucose carbon is diverted to biosynthetic pathways to fuel cell proliferation. Entry of pyruvate into mitochondrial oxidative metabolism is mediated primarily by the PDH complex. In cancer cells, the great majority of pyruvate is converted to lactate and is kept away from mitochondria, which is in part due to inhibition of PDH. This metabolic feature allows cancer cells to evade over-production of ROS that are byproducts of mitochondrial oxidative metabolism, thereby acquiring resistance to pro-oxidant therapies and enhanced metastatic potential. Src and PDK independently inactivate PDH through direct tyrosine (Y) and serine (S) phosphorylation, respectively. Furthermore, ROS may activate Src, resulting in a negative feedback loop to restrain cellular oxidative stress (see discussion). Green arrows indicate the flow of glucose carbon. OXPHOS: oxidative phosphorylation.

Because Src-mediated PDHA1 tyrosine phosphorylation is independent of PDK-mediated serine phosphorylation, we asked whether simultaneous inhibition of Src and PDK activities might additively induce ROS generation. Dichloroacetate (DCA) is a commonly used PDK inhibitor, acting to activate PDH and oxidative metabolism [59–61]. DCA treatment increased ROS levels in 4T1 cells expressing WT PDHA1 (Figure 5A). Compared with WT PDHA1, expression of Src-resistant Y289F PDHA1 also enhanced ROS production. DCA treatment of cells expressing Y289F PDHA1 led to an additive effect on ROS stimulation (Figure 5A). The results are consistent with the model that Src and PDK independently suppress PDH and ROS generation (Figure 5B).

### Src confers resistance to pro-oxidant therapy through suppression of oxidative metabolism

Ionizing radiation and many chemotherapy drugs kill cancer cells by directly or indirectly generating free radicals [6, 8]. Therefore, cancer cells with decreased oxidative stress and/or enhanced antioxidant capacity exhibit intrinsically heightened resistance to pro-oxidants. Because activation of Src reduced PDH activity and ROS levels (Figure 1 and Supplementary Figure S1), we hypothesized that Src's metabolic effect might contribute to therapeutic resistance.

**Figure 6:**
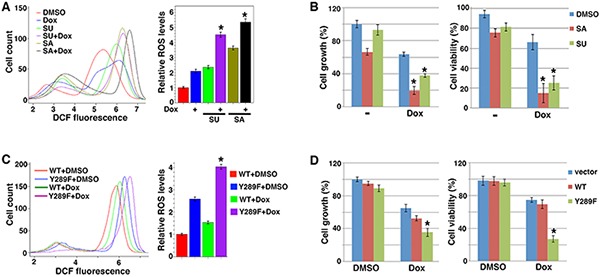
Blocking Src's metabolic effect sensitizes cancer cells to pro-oxidant chemotherapy **A, B.** Src inhibitors and chemodrug Doxorubicin (Dox) show additive/synergistic effects on ROS induction and suppression of cell growth/viability. 4T1 cells were treated with Dox (1 μg/ml) and Src inhibitors SA and SU (1 μM each). ROS levels (A) and cell growth and viability (B) were subsequently determined. **C, D.** Expression of PDHA1 Y289F sensitizes cells to pro-oxidant. 4T1 cells transduced with WT or Y289F PDHA1 were treated with vehicle (DMSO) or Dox, followed by measurement of ROS levels (C) and cell growth and survival (D). Error bars represent S.D. *p < 0.05.

As Src inhibitors SU6656 and Saracatinib activated PDH and stimulated ROS production (Figure 2), we tested whether they could sensitize Src-activated cancer cells to pro-oxidants. Chemodrug Doxorubicin, a topoisomerase II inhibitor, is able to augment ROS generation [6]. We treated 4T1 cells with Doxorubicin and Src inhibitors singly or in combination, and measured ROS levels and cell numbers. Doxorubicin and SU6656 each caused 2-2.5 fold increases in ROS levels, but combination of both drugs led to a nearly 5-fold increase of ROS (Figure 6A). Like SU6656, Saracatinib also exhibited an additive effect on increasing ROS levels when combined with Doxorubicin (Figure 6A). Doxorubicin alone modestly reduced the growth rate and viability of 4T1 cells (Figure 6B). As expected, the Src inhibitors synergized with Doxorubicin to suppress cell growth and survival (Figure 6B). The results show that Src inhibitors can sensitize cancer cells to pro-oxidant chemotherapy.

To verify that Src promoted therapy resistance through its metabolic reprogramming activity, more specifically, PDHA1 Y289 phosphorylation, we compared 4T1 cells expressing WT or Y289F PDHA1 for their sensitivity to Doxorubicin. Y289F PDHA1-expressing cells indeed generated more ROS than cells expressing WT PDHA1 when both were treated with Doxorubicin (Figure 6C). Accordingly, cells expressing PDHA1 Y289F were markedly more sensitive to Doxorubicin than cells expressing WT PDHA1 with regard to cell growth and survival (Figure 6D). Taken together, these results suggest that Src-mediated metabolic regulation through PDHA1 Y289 phosphorylation promotes cancer cell's resistance to pro-oxidant chemotherapy.

## DISCUSSION

The Warburg effect, which describes the uncoupling of glucose oxidation from increased glycolysis in cancer cells, promotes tumor growth, metastasis, and therapy resistance (Figure 5B) [2, 5, 7]. Src is a classical oncogene with well-established pro-malignant functions. However, the potential role of Src in metabolism and its link to the Warburg effect remained largely elusive. Early studies in 1980s reported that cells infected with viral Src showed signs of increased glucose transport and glycolysis [62, 63]. The present study reveals that Src, which is present in mitochondria in addition to cytoplasm [40–47], can inactivate PDH through direct tyrosine phosphorylation, thereby attenuating the flux of glycolysis-derived pyruvate into mitochondrial oxidative metabolism. The function of Src in metabolic regulation thus parallels that of PDKs (Figure 5B). Collectively, these findings uncover Src's metabolic reprogramming role and elucidate a biochemical mechanism by which Src contributes to the Warburg phenotype in cancer cells.

Ionizing radiation and many chemotherapy drugs exert cytotoxic activities in large part by directly or indirectly inducing ROS [6, 8]. Cancer cells with lower levels of oxidative stress can tolerate higher levels of pro-oxidants [7,8, 64]. Because Src can inactivate PDH through tyrosine phosphorylation and attenuate mitochondrial ROS production, Src-activated cancer cells are expected to be intrinsically resistant to pro-oxidant therapy. This is consistent with the fact that Src-activated cancers are generally refractory to conventional therapies. Moreover, Src is a redox-sensing kinase and is activated through direct oxidation of its cysteine residues by ROS [65–69]. When cancers with low Src activity are treated with pro-oxidants, Src may become activated by ROS. Indeed, it was reported that chemodrug Oxaliplatin activated Src through induction of intracellular ROS [70]. ROS-activated Src is expected to in turn inhibit PDH to decrease mitochondrial ROS production. This negative feedback may thus maintain cellular redox homeostasis and enable cancer cells to acquire resistance to pro-oxidants (Figure 5B). Taken together, Src's metabolic effect confers both intrinsic and acquired resistance to pro-oxidant therapy. Combination of Src inhibitors with pro-oxidants may boost ROS generation and lead to synergistic therapeutic effects (Figure 6B).

## MATERIALS AND METHODS

### Cell culture, chemicals, antibodies and constructs

HEK293, SW480, SW620 and 4T1 cells were grown in Dulbecco's modified Eagle's medium (DMEM) supplemented with 10% bovine calf serum. AsPC1 cells were cultured in RPMI-1640 medium with 10% fetal bovine serum (FBS). MCF-10A cells were grown in DMEM/F12 with 5% Horse Serum, 20ng/ml EGF, 0.5 mg/ml Hydrocortisone, 10μg/ml Insulin. Src inhibitors PP2, SU6656 and Saracatinib were purchased from Cayman Chemical, and chemodrug Doxorubicin from Sigma. Mouse and rabbit phospho-tyrosine (P-Tyr-1000 and P-Tyr-100) and PDHA1 monoclonal antibodies were from Cell Signaling. Mouse monoclonal Flag M2 antibody was from Sigma. PDHA1 AAA mutant was created by substituting three serine residues (S293, S300, and S232) with alanine. PDHA1 Y289F mutant was generated by replacing tyrosine 289 with phenylalanine. Mutagenesis was achieved using a method described in QuickChange II Site-Directed Mutagenesis (Stratagene).

### Preparation of mitochondrial extract

Mitochondria were prepared as described [71]. Cells (1×10^8^) were washed once with 10 ml Grinding medium (Sucrose 250 mM, EDTA 2 mM, BSA 1 mg/ml, pH 7.4), and centrifuged for 5 min at 800g at 4°C. The pellet was re-suspended in 1ml Grinding medium, sonicated, and centrifuged for 12 min at 800g at 4°C. The supernatant was collected, followed by immediate centrifuge for 20 min at 8,500g at 4°C. The pellet was re-suspended with 500ul buffer S (Sucrose 150 mM, KCl 40 mM, Tris/HCl 25 mM, BSA 1 mg/ml, pH 7.4), sonicated and then centrifuged 20 min at 10,000g at 4°C. Mitochondrial fractions in the pellet were re-suspended in 100 ml buffer S. Protein concentration in mitochondrial fractions were determined by BCA Protein Assay.

### PDH activity assay

PDH-mediated conversion of pyruvate to acetyl-CoA generates NADH. PDH activity measurement was based on the rate of NADH generation (as described in [72]). Pyruvate decarboxylase activity was assayed immediately after preparation of the mitochondrial extracts, at 30°C with a spectrophotometer set at 340 nm. The assay mixture contained the following: potassium phosphate buffer (pH 8.0), 100 mM; MgCl2, 1 mM; thiamine pyrophosphate, 0.2 mM; NAD, 2.5 mM; cysteine-HCl, 2 mM; pyruvate, 5 mM; Triton X-100, 0.05%; and mitochondrial extract. The reaction was started by addition of 0.15 mM coenzyme A. Reaction rates were linearly proportional to the amount of extract added. Each experiment was carried out in triplicate.

### *In vitro* kinase assay

GST-PDHA1 (WT and mutant) fusion proteins that were conjugated on glutathione beads were incubated with purified Src recombinant enzyme (SignalChem) in the Src assay buffer (Hepes 50mM pH7.5, MgCl_2_ 5 mM, ATP 50 μm, NaCl 150 mM, DTT 1 mM, NP-40 0.02%) for 30 min at 30°C. The GST-beads were washed with co-immunoprecipitation buffer (Tris 20mM PH7.5, NaCl 150mM, NP-40 0.5%). Western blotting was carried out with Rabbit anti- Phospho-Tyrosine (P-Tyr-1000) monoclonal antibody (Cell Signaling).

### Quantification of oxygen consumption rate (OCR) and ROS

Oxygen consumption rates were determined with a Seahorse XF Analyzer. Cellular ROS levels were evaluated with carboxy-H2DCFDA (5-(and-6)-carboxy-2, 7′-dichlorodihydrofluorescein diacetate) from Acros Organics. Oxidation of DCFDA to the highly fluorescent 2,7-dichloro-fluorescein (DCF) is proportionate to ROS generation. Adherent cells were incubated with 5 μM DCFDA for 30 minutes in PBS at 37°C in the dark. Cells were washed with PBS, trypsinized, rinsed, and resuspended in cold PBS. Fluorescence data were acquired within 60 minutes using the BD ACCURI C6 Flow Cytometer (using the FL1 channel). Analysis of data was conducted by FCS Express 4 Flow and RStudio with package FlowCore. Mean fluorescence intensity was used for comparison. Each experiment was carried out in triplicate and results were presented as means ± S.D. for each treatment group.

### Cell viability, anoikis, and tail vein injection

Cell viability was determined based on Trypan blue staining using a Bio-Rad TC20 automated cell counter. Anoikis assay was performed as previously reported [4]: MCF10A and SW620 cells expressing WT or Y289F PDHA1 were cultured in suspension for 2 days before counting viable cells. Experimental metastasis assay was conducted by injecting 5 × 10^5^ 4T1 cells into tail vein of NOD/SCID mice (5-6 weeks old) as previously described [4].

## SUPPLEMENTARY FIGURES


