# Supplementary material for: Src drives the Warburg effect and therapy resistance by inactivating pyruvate dehydrogenase through tyrosine-289 phosphorylation
Source: Oncotarget. 2016 Feb 3;7(18):25113–24. doi: 10.18632/oncotarget.7159 (PMC5041892; doi:10.18632/oncotarget.7159)
Supplement: Supplementary file 1 [file oncotarget-07-25113-s001.pdf]

## SUPPLEMENTARY FIGURES

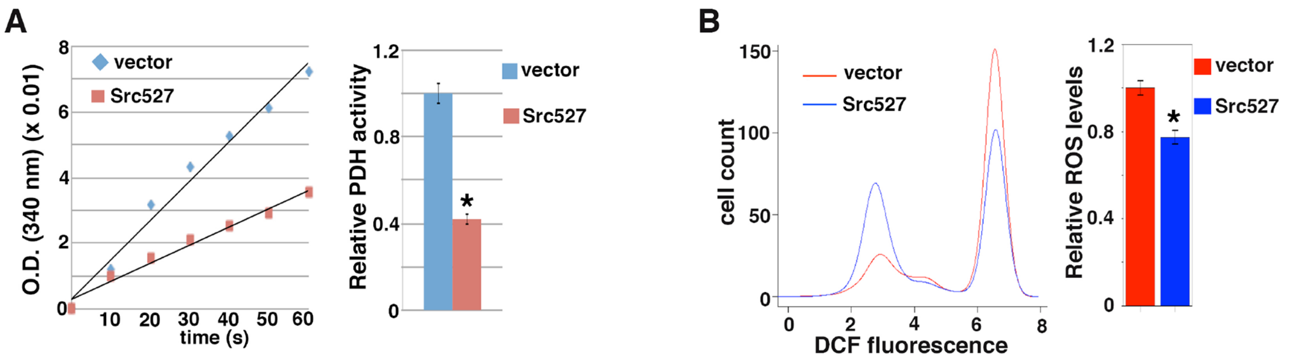


**Supplementary Figure S1: Activation of Src inhibits PDH activity and ROS generation.** SW480 cells were transduced with lentiviral Src527, and subjected to measurement of PDH activity **A.** and ROS levels **B.** Error bars represent standard deviation (S.D.). *$p < 0.05$.

```
PLVLEYETYRYGGHSMSDPGT    budding yeast (Saccharomyces cerevisiae)
PLLMEFVTYRYGGHSMSDPGT    fission yeast (Schizosaccharomyces pombe)
PLVMETNTYRYSGHSMSDPGT    fly (Drosophila melanogaster)
PILMELQTYRYHGHSMSDPGV    human (Homo sapiens)
```

**Supplementary Figure S2: PDHA1 Y289 is highly conserved.** Amino acid sequence alignment of PDHA1 from indicated species (Y289 is in bold and highlighted).

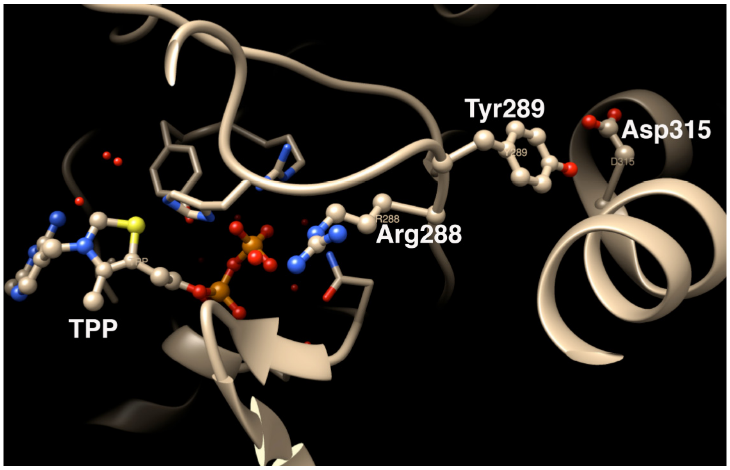


**Supplementary Figure S3: Structural basis for inactivation of PDH by phosphorylation of PDHA1 Y289.** Tyr289 is close to Asp315. Tyr289 phosphorylation causes steric clash between the phosphoryl group and Asp315. The repulsion may push Tyr289 away, and hence affect the positioning of neighboring Arg288, which is an important anchor for TPP.

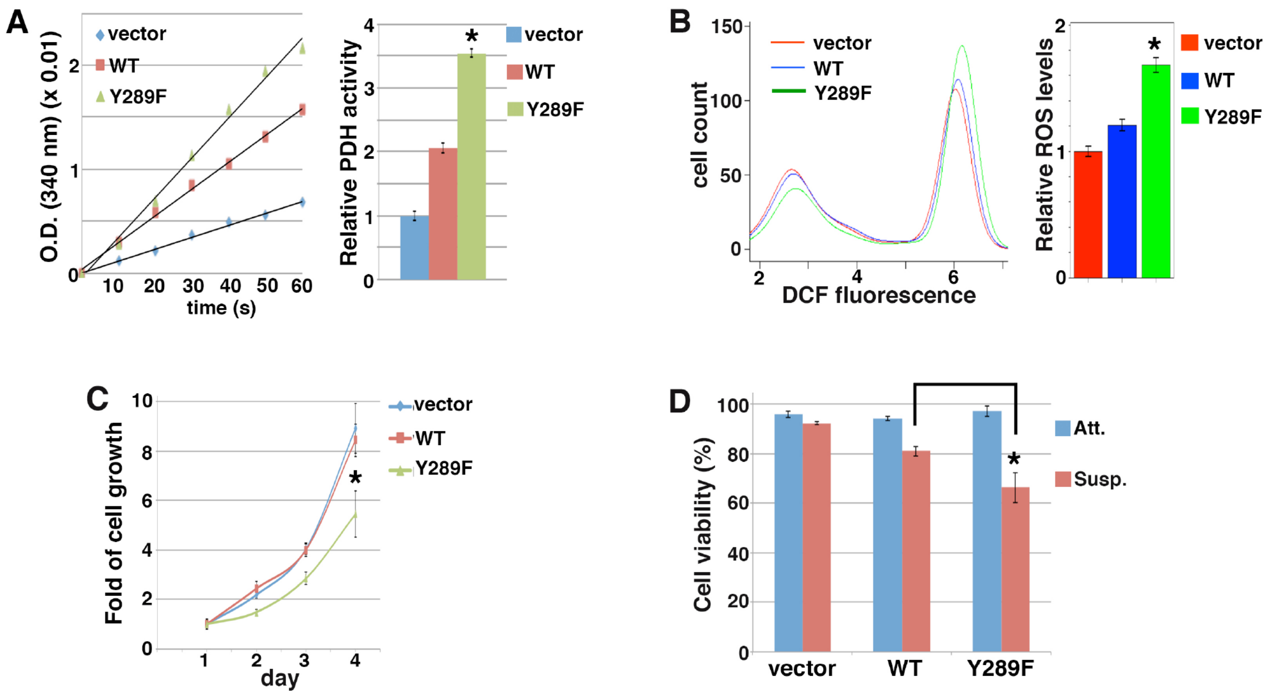


**Supplementary Figure S4: PDHA1 Y289F mutant activates PDH and ROS generation, reduces cell growth and sensitizes cells to anoikis.** SW620 cells were transduced with lentiviral vector or PDHA1 (WT or Y289F), followed by analyses of PDH activity **A.** ROS levels **B.** cell growth **C.** and anoikis **D.** Error bars represent S.D. *p < 0.05 (comparing Y289F with WT).
